# LRRK2 dephosphorylation increases its ubiquitination

**DOI:** 10.1042/BJ20141305

**Published:** 2015-06-19

**Authors:** Jing Zhao, Tyler P. Molitor, J. William Langston, R. Jeremy Nichols

**Affiliations:** * The Parkinson's Institute, 675 Almanor Ave, Sunnyvale, CA 94085, U.S.A.

**Keywords:** kinase, kinase inhibitor, Parkinson's disease, phosphatase, ubiquitin

## Abstract

Activating mutations in the leucine rich repeat protein kinase 2 (*LRRK2*) gene are the most common cause of inherited Parkinson's disease (PD). LRRK2 is phosphorylated on a cluster of phosphosites including Ser^910^, Ser^935^, Ser^955^ and Ser^973^, which are dephosphorylated in several PD-related LRRK2 mutants (N1437H, R1441C/G, Y1699C and I2020T) linking the regulation of these sites to PD. These serine residues are also dephosphorylated after kinase inhibition and lose 14-3-3 binding, which serves as a pharmacodynamic marker for inhibited LRRK2. Loss of 14-3-3 binding is well established, but the consequences of dephosphorylation are only now being uncovered. In the present study, we found that potent and selective inhibition of LRRK2 kinase activity leads to dephosphorylation of Ser^935^ then ubiquitination and degradation of a significant fraction of LRRK2. GNE1023 treatment decreased the phosphorylation and stability of LRRK2 in expression systems and endogenous LRRK2 in A549 cells and in mouse dosing studies. We next established that LRRK2 is ubiquitinated through at least Lys^48^ and Lys^63^ ubiquitin linkages in response to inhibition. To investigate the link between dephosphorylation induced by inhibitor treatment and LRRK2 ubiquitination, we studied LRRK2 in conditions where it is dephosphorylated such as expression of PD mutants [R1441G, Y1699C and I2020T] or by blocking 14-3-3 binding to LRRK2 via difopein expression, and found LRRK2 is hyper-ubiquitinated. Calyculin A treatment prevents inhibitor and PD mutant induced dephosphorylation and reverts LRRK2 to a lesser ubiquitinated species, thus directly implicating phosphatase activity in LRRK2 ubiquitination. This dynamic dephosphorylation–ubiquitination cycle could explain detrimental loss-of-function phenotypes found in peripheral tissues of LRRK2 kinase inactive mutants, LRRK2 KO (knockout) animals and following LRRK2 inhibitor administration.

## Abstract (precis)

LRRK2 is normally phosphorylated at Ser910/935/955/973, but is dephosphorylated in certain PD associated mutations and after kinase inhibition. We ascribe a novel functional significance to the regulation of Ser910/935 as a switch for LRRK2 ubiquitination for downstream signaling and/or degradation.

## INTRODUCTION

Parkinson's disease (PD) is a progressive neurodegenerative disorder with no known cure. PD is typically of idiopathic origin; however, it has been established that environmental exposures to toxins and inheritance of dominant or recessive mutations can precipitate the onset of disease. Autosomal dominant, mis-sense mutations in the leucine rich repeat protein kinase 2 (LRRK2) gene are the most common genetic predisposition to develop PD [1–5]. LRRK2 mutations account for approximately 1%–5% of familial and sporadic PD and are inherited as autosomal dominance with incomplete penetrance [6–11]. Importantly, LRRK2-associated PD closely resembles idiopathic disease clinically but with pleiomorphic pathology, sometimes lacking Lewy bodies [2,3,12]. The most common mutation leads to a serine substitution of Gly^2019^ in subdomain VII of the kinase domain [8], which increases kinase activity 2–4-fold [13–15]. Other pathogenic inherited mutations in the Ras of complex/C-terminal of ROC (Roc/COR) domain (R1441G/C/H, Y1699C and N1437H) have disrupted GTPase activity and increased kinase activity.

In untreated cells and tissues, LRRK2 is basally phosphorylated on Ser^910/935/955/973^, referred to herein as the upstream kinase sites. These serines are regulated by upstream kinases and phosphatases in a manner dependent on LRRK2 kinase activity [16]. LRRK2 is rapidly dephosphorylated at the autophosphorylation site (Ser^1292^) and upstream kinase phosphosites after inhibition. Dephosphorylation of the upstream kinase sites and loss of 14-3-3 phospho-dependent binding has therefore been a reliable indicator of inhibition of LRRK2 kinase activity. Acute inhibition of ectopically expressed LRRK2 drives its relocalization in cells to inclusions or accumulations. Pathogenic PD mutations (N1437H, R1441C/G, Y1699C and I2020T) have increased kinase activity, but are dephosphorylated at the upstream kinase sites and form cytoplasmic accumulations and filamentous skein like structures with an overall reduction in stability [17–22]. G2019S pathogenic mutation exhibits increased kinase activity and is phosphorylated to wild-type (WT) levels at Ser^910/935/955/973^. A unifying theme between inhibition and N1437H, R1441C/G, Y1699C and I2020T mutations is lack of upstream kinase phosphorylation and a demonstrated lack of stability in cell systems and accumulation in cytoplasmic inclusions [18,21–23]. The nature of these inclusions has yet to be determined, but can be vesicular and/or microtubule linked [19,24].

It is postulated that increased LRRK2 activity leads to PD, therefore strategies that safely reduce LRRK2 kinase activity may be of therapeutic benefit in genetic PD. In support of this hypothesis, the *substantia nigra* of LRRK2 knockout (KO) rat is protected from immune insult with lipopolysaccharide or viral α-synuclein overexpression [25]. Cultured LRRK2 homozygous KO cells also show increased neurite length and branching [26,27]. However, the peripheral tissues of LRRK2 homozygous KO animals develop accumulations of lamellar bodies and lipofuscin positive inclusions in lung and kidney respectively [27–32]. Animals harbouring a knock-in kinase-dead (KD; D1994S) LRRK2 allele also show pathology in the kidney and reduced LRRK2 protein levels [29]. Non-human primates (NHP, *Cynomolgus*) treated with two structurally distinct LRRK2 inhibitors for up to 1 month developed similar lamellar body formation in the lung and decreased LRRK2 in the lung of treated animals [33]. LRRK2 inhibitors may protect the brain, but at the expense of inducing pathology in the periphery. To date, no animal model has been developed that induces a robust parkinsonian phenotype dependent on LRRK2 expression or kinase activity, precluding elucidation of neuroprotective concentrations of LRRK2 inhibitor that would not induce peripheral phenotypes. The importance of defining the molecular consequences of LRRK2 inhibition is elevated due to this lack of a therapeutic window for LRRK2 drugs that reduce neuropathology and disease while avoiding peripheral effects.

Dephosphorylation of Ser^910/935^ is a common outcome measure for kinase inhibition of LRRK2 and also the PD-related mutations N1437H, R1441C/G, Y1699C and I2020T. We propose that inhibition of LRRK2 kinase activity and dephosphorylation of Ser^910/935/955/973^ results in the ubiquitination and degradation of LRRK2. In the present study, we provide evidence of a molecular model in support of this hypothesis. Selective inhibition reduces the accumulation of LRRK2 in a variety of cell and tissue models. We found that dephosphorylation of the LRRK2 Ser^935^ after LRRK2 inhibition increases ubiquitination of LRRK2 through Met^1^, Lys^48^ and Lys^63^ linkages and probably other linkage sites. It is therefore likely that full repression of kinase activity with small molecules could result in detrimental effects associated with the absence of LRRK2. These results suggest that complete inhibition of LRRK2 kinase activity could not only suppress mutation-induced neurodegeneration, but also cause detrimental loss-of-function phenotypes in peripheral tissues. These conclusions predict that targeting the activated LRRK2 G2019S may be a better approach for PD caused by this mutation. If over-activation of LRRK2 causes idiopathic PD, a selective but lower potency compound may be useful. Finally, identifying ubiquitin ligases and deubiquitinases (DUBs) that act on LRRK2 will be crucial to understanding the full gamut of LRRK2 signalling.

## MATERIALS AND METHODS

### Buffers, chemicals and antibodies

Lysis buffer contained 50 mM Tris/HCl, pH 7.4, 1 mM EGTA, 1 mM EDTA, 1 mM sodium orthovanadate, 10 mM sodium β-glycerophosphate, 50 mM NaF, 5 mM sodium pyrophosphate, 0.27 M sucrose, 1 mM benzamidine and 1 mM PMSF and was supplemented with 1% Triton X-100 and 10 mM N-ethylmaleimide (NEM). Buffer A contained 50 mM Tris/HCl, pH 7.4, 50 mM NaCl, 0.1 mM EGTA and 0.27 M sucrose. LRRK2 kinase inhibitor GNE1023 was described in [34] and synthesized at Genentech; LRRK2-IN1 and small molecule enhancer of rapamycin 28 (SMER28) were purchased from Tocris. Non-selective, reversible inhibitor of DUBs and ubiquitin-like isopeptidases, PR-619, was purchased from LifeSensors. Anti-GFP (clones 7.1 and 13.1) and anti-HA (haemagglutinin; clone 3F10) antibodies are from Roche. Anti βIII-tubulin (TU-20), actin (D6A8), LC3B (D11), p53 (1C12), total ubiquitin (P4D), ubiquitin Lys^48^ (D9D5) and ubiquitin Lys^63^ (D7A11) are from Cell Signaling Technology. Anti LRRK2 (N241) is from Neuromab. Anti-LRRK2 pS935 (UDD2 [12]) and anti-LRRK2 (UDD3) were obtained from the Division of Signal Transduction Therapy (DSTT) or along with anti-ubiquitin Lys^48^ (EP8589) were from Epitomics. Anti-LRRK2 pS1292 was generously provided by Genentech. Anti p62 (5F2) is from MBL. The ubiquitin antibody (FK2) used in the immunofluorescence is from Enzo. Anti-total ubiquitin (VU-1) is from life sensors. Anti-ubiquitin Lys^63^ (Apu3) is from Millipore. 14-3-3 overlay was carried out as described in [17].

### Cell culture, treatments and cell lysis

Tissue culture reagents were from Life Technologies or Thermo Scientific. Human embryonic kidney (HEK)-293 cells were cultured in Dulbecco's Modified Eagle's medium (DMEM) supplemented with 10% FBS, 2 mM glutamine and 1× antimycotic/antibiotic solution. The Flp-in T-REx system was from Invitrogen and stable cell lines were generated as per manufacturer instructions by selection with hygromycin as has been described previously [17,18]. T-REx cell lines were cultured in DMEM supplemented with 10% FBS and 2 mM glutamine, 1× antimycotic/antibiotic and 15 μg/ml blasticidin and 100 μg/ml hygromycin. GFP-tagged LRRK2 lentivirus stable transduced SH-SY5Y cells were generated as described [35,36]. SH-SY5Y cells were maintained in 1:1 DMEM:F-12 and MEM with L-glutamine and 10% FBS. Human lung alveolar epithelial A549 cells were cultured in DME/F-12 with L-glutamine and 10% FBS, 1× antimycotic/antibiotic.

HEK293 and T-REx were transfected by the polyethylenimine method [37]. T-REx cultures were induced to express the indicated protein by inclusion of 1 μg/ml doxycycline in the culture medium for 24–48 h. Cells transfected with WT and mutant LRRK2 plasmids were lysed 48 h after transfection. A549 cells were transfected with Lipofectamine LTX. Cell treatments were added at the indicated time and concentration. After the indicated culture conditions, cell lysates were prepared by washing once with PBS and lysing in situ with 0.5 ml of lysis buffer per 10 cm dish on ice, then centrifuged at 15000 ***g*** at 4°C for 15 min. Protein concentrations were determined using the Bradford method with BSA as the standard. Terminal SH-SY5Y differentiation was performed essentially as described [38]. SH-SY5Y cells were grown in medium containing 10 μM retinoic acid (RA) for 3 days; then the medium was removed and replaced with fresh in 80 nM TPA for another 3 days of differentiation.

### DNA constructs

Restriction enzyme digests, DNA ligations and other recombinant DNA procedures were performed using standard protocols with Fermentas or LifeTechnologies enzymes. DNA constructs used for transfection were purified from *Escherichia coli* DH5α using Qiagen plasmid Maxi kits or Invitrogen Maxi prep kits according to the manufacturer's protocol. The pcDNA5–Frt–Flag–LRRK2 and pcDNA5–Frt–GFP LRRK2 constructs used for transfections were provided by Dr Dario Alessi (MRC-PPU, University of Dundee, U.K.). The Difopein expression construct was generated by ligating a codon optimized difopein coding sequence to pcDNA5–Frt–GFP vector (synthesized by Life Technologies) [39]. pRK5–HA–ubiquitin WT, Lys^48^ and Lys^63^ linkage plasmids were a kind gift of Dr Ted Dawson [40] and obtained from Addgene. N-terminal methionine mutants of ubiquitin, WT M1L, Lys^48^ M1L, Lys^63^ M1L and Lys^0^ M1L were generated by GeneArt Site-Directed Mutagenesis system (Life Technologies). All DNA constructs were verified by DNA sequencing, performed by Sequetech.

### LRRK2 immunoprecipitation assays

For transfected HEK293 or T-REx cells, cell lysates were prepared in lysis buffer (0.5 ml per 10-cm dish) and subjected to immunoprecipitation with anti-FLAG M2 agarose (Sigma) or GFP-Trap A beads (Chromotek) at 4°C for 1 h. Beads were washed twice with lysis buffer supplemented with 300 mM NaCl and then twice with buffer A. Immune complexes were incubated at 70°C for 10 min in lithium dodecyl sulfate (LDS) sample buffer, passed through a Spin-X column (Corning) to separate the eluate from the beads, then boiled. The eluates were subjected to Western blots with indicated antibodies. For endogenous immunoprecipitation assays, LRRK2 was immunoprecipitated using anti-LRRK2 (UDD3; DSTT, MRC-PPU, Dundee University) non-covalently conjugated to protein-A sepharose (1μg of antibody:1 μl of bead) and incubated at 4°C for 4 h and analysed by immunoblotting, as indicated.

### Immunofluorescence

A549 cells were plated in eight-well glass bottom, CC2™ coated chamber slides (Nunc). One-day after plating, the cells were transfected with GFP tagged LRRK2 WT or mutants (S910/935A, R1441G, I1699C, G2019S and I2020T) and/or HA–ubiquitin (WT, Lys^48^ or Lys^63^). Twenty-four hours after transfection, the cells were treated with DMSO or 2 μM GNE1023 for 24 h. The cells were fixed in 4% formaldehyde buffered in PBS (Electron Microscopy sciences). Cells were permeabilized in 0.5% Triton X-100 in PBS for 5 min, blocked with 10% goat serum and stained with indicated primary antibodies in 3% goat serum, at 4°C for 18 h. Images were taken on a Nikon TiE microscope with a 60× long working distance objective and representative images are shown. Z-stacked images were captured in 0.5 micron steps. Deconvolved images were generated using the 3D Landweber deconvolution method on NIS elements platform and are shown in a maximal projection image.

### Quantitative real-time PCR

A549 cells were treated with DMSO or 5 μM GNE1023 for 48 h. Total RNA was isolated with PureLink™ RNA Mini Kit and RNAs were treated with PureLink™ DNase (Ambion, Life Technologies). The first strand cDNA synthesis was carried out with ReadyScript cDNA Synthesis Mix (Sigma). The Taqman probes used in quantitative real-time PCR are from Life Technologies, human LRRK2 primer 1, Hs00968202_m1, LRRK2 primer 2, Hs00968209_m1 and LRRK2 primer3, Hs00968191_m1, Mouse Lrrk2 primer 1, Mm01304127_g1 and Lrrk2 primer 2, Mm00481934_m1. Quantitative real-time PCR was performed with TaqMan Fast advanced Master Mix (Life Technologies) and the signals were detected in a BioRad CFX96 Real-Time System/C1000 Thermal Cycler. The fold difference in gene expression was calculated using the comparative Ct method (2^−ΔΔCt^) by Bio-Rad CFX manager 3.1 and gene expression was normalized to the housekeeping gene ACTB for human, and Tbp (Mm00446971_m1) and Hprt (Mm01545399_m1) for mouse.

### LRRK2 inhibitor treatment of mice

GNE1023 selectivity was assessed in the Life Technologies panel of 256 kinases. WT 1-year-old FVB/N laboratory strain of mice from Jackson Labs were maintained and treated under the approval of the Parkinson's Institute Institutional Animal Care and Use Committee. Mice were treated with 100 mg/kg GNE1023 suspended in a 0.1% Avicel solution by oral gavage or vehicle alone. At 6 h post administration, animals were sacrificed by cervical dislocation in accordance with IACUC approved protocols, and organs were harvested and massed. Homogenates were made with a rotary homogenizer in 5× tissue mass:volume of lysis buffer containing, Sigma protease cocktail, 1 mM PMSF, 1 mM benzamidine, 1% TritonX-100/0.1% SDS. Soluble protein was separated by sequential centrifugation at 800 ***g***/4°C then 15000 ***g***/4°C and 50 μg of total soluble protein was analysed by immunoblotting.

### Quantification, statisticsal analysis and image processing

Quantification of Western blot intensity was performed by ODYSSEY infrared imaging system application software LI-COR, version 3.0.30. The mean values of the intensities were graphed in GraphPad Prism 6, with the S.E.M. LRRK2 aggregate induction by ubiquitin and ubiquitin mutant co-expression were quantified by counting the ratio of the LRRK2 aggregates in the total number of co-transfected cells (GFP–LRRK2 and Alexa594-Ubiquitin). The mean values were graphed in GraphPad Prism6 with S.E.M. and significance was determined by the Chi-square of the total counts of LRRK2 aggregates. Half-life determinations were performed as previously described for LRRK2 [41]; LRRK2 protein levels were graphed in GraphPad Prism for non-linear curve fit analysis. Estimated half-lives are presented with 95% confidence intervals (CIs). All the images were processed in Adobe Photoshop CS4, version 11.0.2 and figures were generated in Adobe illustrator CS4, 14.0.0.

## RESULTS

### Inhibition of LRRK2 decreases its accumulation in cells and in tissues

**Figure 1:**
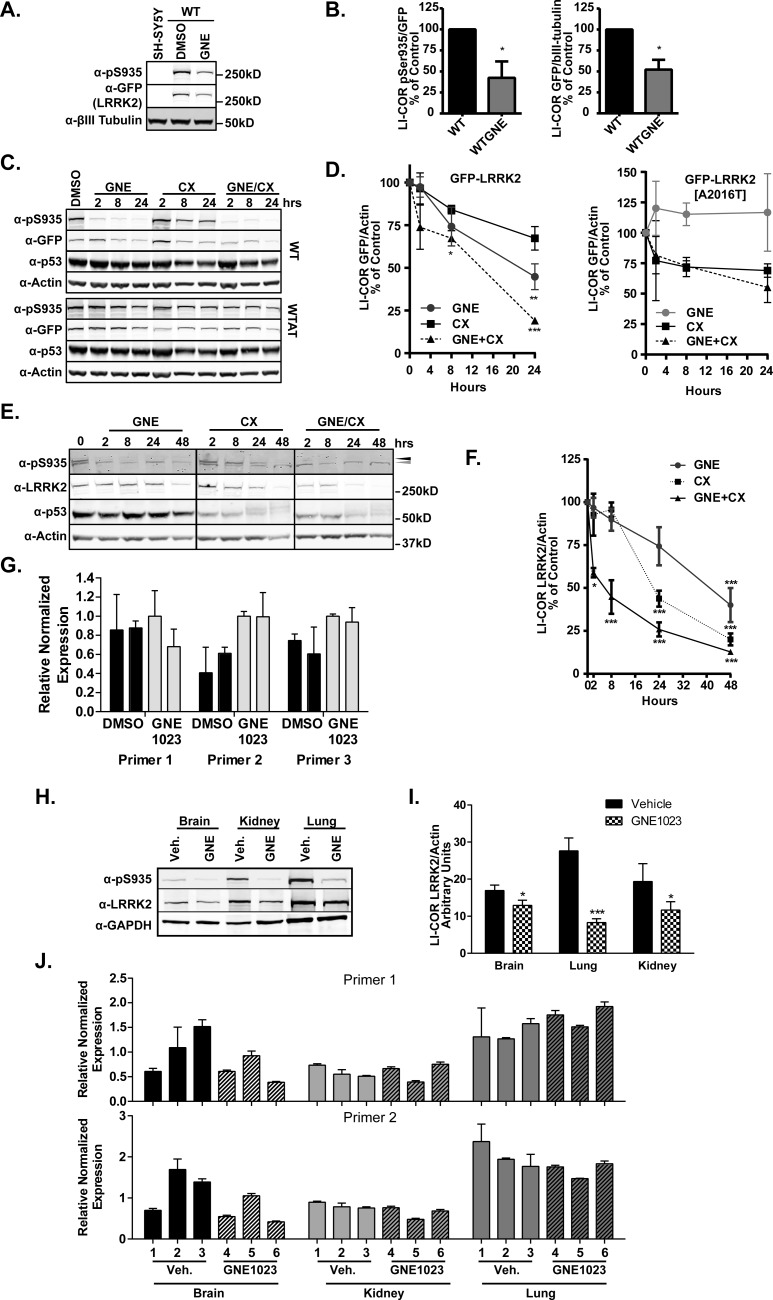
LRRK2 kinase inhibition decreases LRRK2 accumulation (**A**) Differentiated SH-SY5Y cells stably expressing GFP–LRRK2 were treated with DMSO or 2 μM GNE1023 for 24 h. SDS-soluble (0.5%) lysates were immunoblotted for GFP (LRRK2), pSer935 (UDD2) and βIII-tubulin (*n*>3). (**B**) LI-COR quantified values set to untreated control from (**A**) α-GFP/α-βIII-tubulin and α-pSer935/α-GFP (UDD3); one sample *t* test was set to the hypothetical value of 1, *P<0.05. (**C**) HEK293 T-REx cells with inducible GFP–LRRK2 or GFP–LRRK2–[A2016T] were treated with DMSO, 20 μg/ml cycloheximide and/or 2 μM GNE2013 for 24 h and immunoblotted as in (**A**) (*n*=3). (**D**) LI-COR quantified values from (**C**) for GFP–LRRK2 and GFP–LRRK2–[A2016T], α-GFP/α-actin set to DMSO control, mean ± S.E.M., one-way ANOVA, *P<0.01, **P<0.001, ***P<0.0001. (**E**) Human lung epithelial A549 cells were treated with DMSO, 5 μM GNE1023 and/or 20 μg/ml cycloheximide for indicated time points. SDS-soluble (0.5%) lysates were immunoblotted for LRRK2 (N241) and pSer935 (UDD2; grey arrowhead=non-specific, black arrowhead=pSer935) and actin (*n*=4). (**F**) LI-COR quantified values from (**E**) set DMSO treated control, mean ± S.E.M. one-way ANOVA, *P<0.01, **P<0.001, ***P<0.0001. (**G**) Quantitative real-time PCR of GNE2013 treated A549 cells as in (**E**); cells were treated with DMSO or 5 μM of GNE for 48 h in duplicate. Quantitative TaqMan real-time PCR was carried out with three different Taqman LRRK2 probe sets and corrected to *ACTB*, as described in the ‘Materials and Methods’. (**H**) 1yo FVB/N mice were administered GNE2013 (100 mg/kg; *N*=7) or 0.1% Avicel vehicle control (*n*=6) for 6 h and 1% TX-100/0.5% SDS soluble whole brain, lung and kidney tissue lysates were prepared. Lysates were immunoblotted for LRRK2 (N241), pSer935 (UDD2) and actin. (**I**) LI-COR quantified values from (**H**), mean ± S.E.M., *t* test, *P<0.05, ***P<0.0005. (**J**) Quantitative real-time PCR of brain, kidney and lung of GNE2013 dosed mice from in (**H**), was carried out with two Taqman LRRK2 probe sets (*n*=3 each, vehicle and control).

Inactivating mutations of LRRK2 kinase activity have been observed to result in lower steady-state accumulation of LRRK2 [29]. We reasoned that there must be a kinase-dependent signal from LRRK2 that decreases its accumulation. We postulated that long-term treatment of LRRK2 inhibitors (24+ h), would mimic the lack of accumulation similar to the kinase inactive LRRK2 observations. GNE1023 was disclosed as a selective inhibitor of LRRK2 [34], we employed this molecule in these studies and show in Supplementary Table S1, the profile of inhibition against 256 kinases, confirming its highly selective activity. We examined the steady state accumulation levels of LRRK2 in differentiated SH-SY5Y cells stably expressing GFP–LRRK2 [42] treated with GNE1023 [34] for 24 h and found a significant diminution of LRRK2 levels to half that of DMSO-treated cells, (GFP/βIII-tubulin) Figures 1(A and B). This decreased accumulation is accompanied by Ser^935^ dephosphorylation, which is a pharmacodynamic marker for LRRK2 inhibition (pSer^935^/GFP; Figures 1A and B).

We next examined the direct role of inhibiting LRRK2 kinase activity on the stability of GFP–LRRK2 and GFP–LRRK2–[A2016T] protein expressed in HEK293 T-REx cells. The A2016T mutation of LRRK2 retains kinase activity but is desensitized to small molecule inhibition *in vitro* and in cells [16,43–47]. Therefore, if inhibition of LRRK2 kinase activity leads to decreased accumulation, then the A2016T mutant should be refractory to inhibitor with no change in accumulation. To investigate if inhibition changes the half-life of LRRK2 over time, we evaluated LRRK2 protein levels in T-REx cells induced to express LRRK2 or LRRK2–[A2016T] for 24 h, then chased into medium containing GNE1023 with or without cycloheximide for the indicated time-points (Figure 1C) and quantified in Figure 1(D). GNE1023 treatment caused a significant decrease in WT LRRK2 stability to approximately half that of untreated LRRK2, 19.91 h (95% CI of 14.02–34.32 h). LRRK2–[A2016T] is not less stable after treatment with GNE1023 showing no change in accumulation at 24 h of inhibitor treatment compared with WT LRRK2. Cycloheximide treatment of GFP–LRRK2 or GFP–LRRK2–[A2016T] cells resulted in an estimated half-life of 40 h, (Figures 1C and 1D). Co-treatment of cells with GNE1023 and cycloheximide significantly affected LRRK2 stability, decreasing the half-life to 11.7 h (95% CI of 8.02–21.58 h). Meanwhile, LRRK2–[A2016T] showed no significant difference in response to cycloheximide treatment alone or with GNE1023 (Figures 1C and 1D). This confirms that inhibition of LRRK2 kinase activity influences the stability of LRRK2. LRRK2 inhibition was maintained with 2 μM GNE1023 for the time-course, as shown by pSer935 immunoblot. These data directly show that kinase inhibition decreases the stability of LRRK2.

We next asked if inhibiting LRRK2 with GNE1023 decreased the stability of endogenous LRRK2 by analysing physiological levels of inhibited LRRK2 in human lung epithelial A549 cells. Homozygous LRRK2 KO in mice and rats results in the accumulation of lamellar bodies of the type II pneumocytes of the lung and A549 can serve as an *in vitro* model of these cells [49] while also expressing LRRK2 [50]. We compared the half-life of LRRK2 after treatment with GNE1023, cycloheximide or both compounds for 2, 8, 24 and 48 h (Figure 1E) and quantified in Figure 1(F). We observed sustained Ser^935^ dephosphorylation and significant decrease in LRRK2 levels at 48 h of GNE1023 treatment with a half-life of 41.07 h (95% CI of 29.51–67.53 h). The half-life of LRRK2 in the presence of cycloheximide was 21.89 h (95% CI, 16.35–31.93 h), which confirms previous findings as in [48]. However, inhibition of LRRK2 and protein synthesis reduced the half-life of LRRK2 more than that of either treatment alone to 11.05 h (95% CI, 7.66–19.81 h; (Figure 1E) and quantified in Figure 1(F). Suppression of p53 stability is complete in these cells as they contain unmutated p53 compared with HEK293, where slight decrements in p53 are seen with cycloheximide (Figure 1E). No change in endogenous LRRK2 mRNA levels at 48 h of GNE1023 treatment (Figure 1G) confirms the effect is at the protein level.

We extended our analysis of LRRK2 to an *in vivo* setting by dosing 1-year-old mice with GNE1023. This compound is blood–brain barrier penetrant and engages LRRK2 in brain and the peripheral organs [34]. With a 100 mg/kg dosing, we analysed LRRK2 protein levels in kidney, lung and brain at 6 h after administration. We observed a GNE1023-dependent decrease in LRRK2 protein levels to 20% of vehicle treated mice in lung, 60% in kidney and 80% in brain (Figure 1H) and quantification shown in Figure 1(I). Analysis of LRRK2 mRNA levels with two different primer probe sets showed no difference in LRRK2 expression, confirming the effect is at the protein level (Figure 1J).

### LRRK2 inhibition is linked to increased ubiquitination

**Figure 2:**
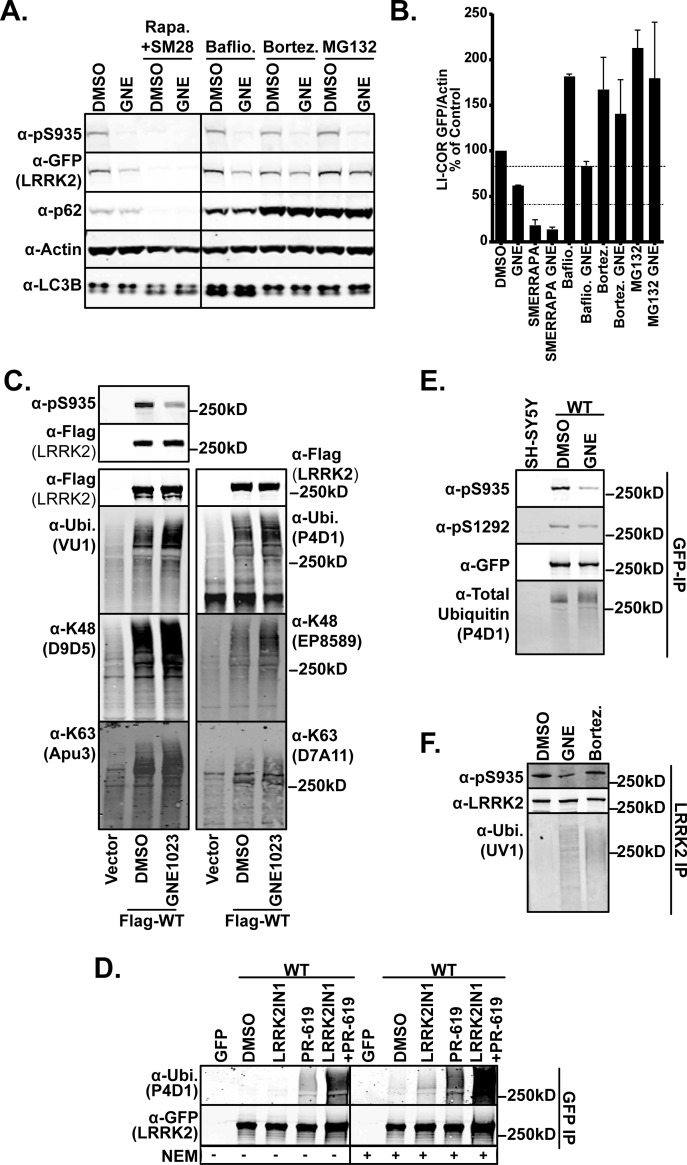
Inhibition of LRRK2 leads to increased ubiquitination (**A**) SH-SY5Y cells stably expressing GFP–LRRK2 were treated with rapamycin (1 μM)/SMER28 (50 μM) for 48 h. Bafilomycin (400 nM), Bortezomib (100 nM), MG132 (5 μM) and/or GNE1023 (2 μM) were added 24 h before harvest. SDS-soluble (0.5%) lysates were immunoblotted for GFP (LRRK2), pSer935 (UDD2), p62 and actin (*n*=2). (**B**) LI-COR quantified values from (**A**), mean ± S.E.M. (**C**) HEK293 cells transfected with vector or FLAG–LRRK2 were treated with DMSO or 2 μM GNE1023 for 24 h. Anti-Flag–M2 immunoprecipitates were immunoblotted with anti-total ubiquitin (VU1 and P4D1), Lys^48^ linkage (D9D5 and EP8589) and Lys^63^ linkage (Apu3 and D7A11). Anti-Flag show equal loading and anti-pSer935 (UDD2) immunoblot shows the decreased phosphorylation by inhibitor treatment. (**D**) T-REx cells expressing GFP or GFP–LRRK2 were treated with DMSO, LRRK2–IN1 (2 μM) for 90 min and PR-619 (50 μM) was added 30 min before harvest where indicated. Samples were lysed in the presence or absence of 10 μM NEM, followed by GFP–Trap immunoprecipitation. Immunoprecipitates were immunoblotted for GFP (LRRK2) and ubiquitin (P4D1). (**E**) Differentiated SH-SY5Y cells with or without stable WT GFP–LRRK2 expression were treated with GNE1023 (2 μM) for 24 h. GFP–Trap-A immunoprecipitates were immunoblotted with pSer935 (UDD2), GFP (LRRK2), pSer1292 and ubiquitin (P4D1). (**F**) A549 cells were treated with 2 μM GNE1023 or 100 nM bortezomib for 24 h. Endogenous LRRK2 was immunoprecipitated (UDD3) and analysed for ubiquitination (UV1), LRRK2 (N241) and pSer935 (UDD2).

From the above data, we conclude that LRRK2 inhibition results in a diminution of protein accumulation in cells and tissues. LRRK2 degradation has been linked to proteosomal and lysosomal routes of proteolysis [48,51,52]; we next examined these pathways in the degradation of LRRK2 in differentiated SH-SY5Y cells stably expressing GFP–LRRK2. We tested if LRRK2 inhibition induced degradation was affected by altering autophagy induction with rapamycin/SMER28 [53–56] or autophagy progression by blockade with the lysosomotropic agent bafilomycin A [57], which prevents the acidification of the lysosome and fusion with the autophagosome; or by proteasome inhibition with two distinct proteasome inhibitors, bortezomib and MG132. Treatment of SH-SY5Y cells expressing GFP–LRRK2 with rapamycin/SMER28 induced the degradation of LRRK2 regardless of inhibitor treatment (Figure 2A) with quantification in Figure 2(B), which is in line with previous data indicating LRRK2 as an autophagy substrate [48,58]. Blocking autophagy or the proteasome increased the steady state levels of LRRK2, similar to [48]. Blocking lysosomal degradation with bafilomycin A did not affect the decrease in LRRK2 accumulation caused by inhibition. However, treatment with proteasome inhibitors reversed the GNE1023-induced reduction in LRRK2 accumulation (Figure 2A) indicating a proteosomal route of degradation. Rapamycin/SMER28 induced the conversion of microtubule-associated protein 1 light chain 3 beta I (LC3I) to LC3II indicating an up-regulation of autophagosome formation. The autophagy cargo protein sequestosome 1 (p62/SQSTM1), which is itself a substrate of autophagy [59], decreased after autophagy induction. Bafilomycin A induced p62 and LC3 accumulation indicating a blockade of autophagy progression. Proteasome inhibition caused a slight induction of LC3 conversion and accumulation [60,61] (Figure 2A).

Together, these data indicate inhibition of LRRK2 results in a decrease in protein stability in cells that is prevented by proteasome inhibition. Since proteasome degradation of proteins is driven by ubiquitination, we next asked if LRRK2 inhibitors triggered ubiquitination of the molecule as it has been shown to be ubiquitinated previously [51,52,62]. We treated HEK293 cells expressing FLAG–LRRK2 with GNE1023 for 24 h then analysed equal amounts of LRRK2 immunoprecipitates by immunoblot with anti-ubiquitin antibodies (Figure 2C). We found that two different antibodies against total ubiquitin (VU1 and PD41), Lys^48^-linked ubiquitin (D9D5 and EP8589) and Lys^63^-linked ubiquitin (Apu3 and D7A11) specifically recognized ubiquitinated LRRK2 (Figure 2C). We enhanced ubiquitination globally by treatment with the broad-based DUB inhibitor PR619 and analysed equal amounts of immunoprecipitates of inhibited LRRK2 in T-REx–GFP–LRRK2 expressing cells. LRRK2 immunoprecipitates show a marked increased ubiquitination after inhibition that is enhanced by PR619 treatment (Figure 2D). Inclusion of NEM in the lysis buffer, which globally alkylates cysteines, inactivating the active-site cysteine of DUBs, enhances detection of LRRK2 ubiquitination. When we analysed the ubiquitination status of LRRK2 from SH-SY5Y neuroblastoma cells stably expressing GFP–LRRK2 treated with GNE1023 for 24 h, we also found that LRRK2 ubiquitination was enhanced by inhibition with GNE1023 (Figure 2E) and confirmed that LRRK2 ubiquitination also occurs in a neuronal background. GNE1023 treatment increased the amount of endogenous ubiquitin detected in immunoprecipitates (Figure 2F), revealing induction of LRRK2 ubiquitination occurs on physiological levels of LRRK2. As a control to detect increase in endogenous LRRK2 ubiquitination, we employed MG132, as in Figure 2(A) and observed increased ubiquitination similar to GNE1023 treatment (Figure 2F).

### Types of ubiquitin linkages on LRRK2

**Figure 3:**
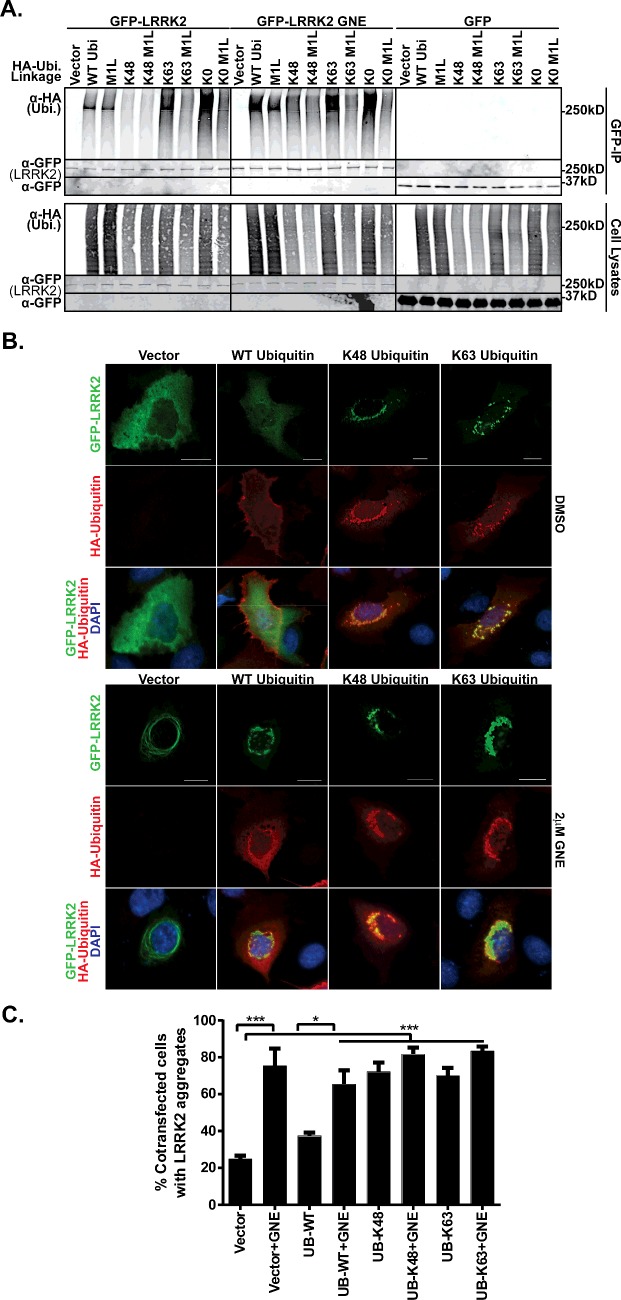
LRRK2 ubiquitination linkage analysis (**A**) HA vector or HA-ubiquitin or the indicated HA-ubiquitin mutants were transfected into HEK293 T-REx–GFP or GFP–LRRK2 expressing cells. Forty-eight hours after transfection, cells were treated with DMSO or 2 μM GNE1023 for 90 min and lysed in buffer containing 10 μM NEM. GFP–Trap-A immunoprecipitates and cell lysate samples were analysed by immunoblot. Anti-HA antibody indicates the conjugation of ubiquitin mutants on LRRK2. Anti-GFP antibody shows equal loading of samples. (**B**) A549 cells transfected with GFP–LRRK2 and HA vector, HA-ubiquitin or the indicated HA-ubiquitin mutants for 24 h and then treated with 2 mM GNE1023 for 24 h. Paraformaldehyde fixed cells were stained with HA (Alexa 594). Cells were imaged for GFP (LRRK2) green and HA (ubiquitin) red and DNA (DAPI) blue. Scale bar is 20 μm. (larger images provided in Supplementary Figure 3). (**C**) Percentage of co-transfected (GFP positive and HA positive) cells with LRRK2 cytoplasmic accumulations, mean ± S.E.M, chi-squared test *P≤0.05, **P≤0.005, ***P≤0.0005. (*n*=4, with at least 25 cells counted per experiment).

Ubiquitin is a diverse signalling molecule in which specific linkages can encode different downstream biological repercussions. The roles of Lys^48^ and Lys^63^ linkages in driving protein degradation and signal transduction are well characterized, whereas the roles of other atypical linkages are now being unravelled [63]. In Figure 2, we observed immunoreactivity of anti-Lys^48^ and -Lys^63^ ubiquitin antibodies on endogenous linkages in LRRK2 immunoprecipitates. To provide support for these linkages on LRRK2, we used an expression system with ubiquitin mutants that allow conjugation through only one lysine residue, Lys^48^ or Lys^63^, whereas all other lysines are mutated to arginine. We found that HA-tagged WT and both Lys^48^ and Lys^63^ ubiquitin linkages could be detected in GFP–LRRK2 immunoprecipitates and both of these linkages increased with inhibitor treatment (Figure 3A). Mutation of all ubiquitin lysines to arginine (Lys^0^) still resulted in ubiquitin conjugation to LRRK2, which was further reduced by mutation of the initiating methionine to leucine (Lys^0^/M1L). Introduction of this mutant into Lys^48^ and Lys^63^ mutants (Lys^48^/M1L and Lys^63^/M1L) reduced the conjugation of ubiquitin to LRRK2, but not other proteins in the cell lysate.

We also asked if ubiquitin and LRRK2 could be found coincidently by immunocytochemistry. WT ubiquitin showed no effect on LRRK2 subcellular localization, whereas expression of the Lys^48^ and Lys^63^ only mutants drove LRRK2 to discrete cytoplasmic locales with skein structures and puncta (Figure 3B) and quantified in Figure 3(C). Expression of the Lys^48^ and Lys^63^ mutants significantly increased the percentage of LRRK2 expressing cells with GFP positive cytoplasmic accumulations to similar levels seen with LRRK2 inhibitor (Figure 3C). We found little co-localization of WT HA-ubiquitin with LRRK2 but that expression of Lys^48^ and Lys^63^ ubiquitin increased HA-ubiquitin positive cytoplasmic accumulations of LRRK2 and co-localized in several instances in the presence and absence of inhibitor, indicated with arrows in Figure 3(B). This outcome is not unexpected, as co-localization of LRRK2 aggregates with ubiquitin has been observed elsewhere as well [34,64]. Further, in a Tau [P30L] and superoxide dismutatase (SOD) A4V expression system with the same ubiquitin constructs used here, Lys^48^ and Lys^63^ co-expression increased ubiquitin positive Tau and SOD inclusions [65].

### LRRK2 dephosphorylation leads to ubiquitination

**Figure 4:**
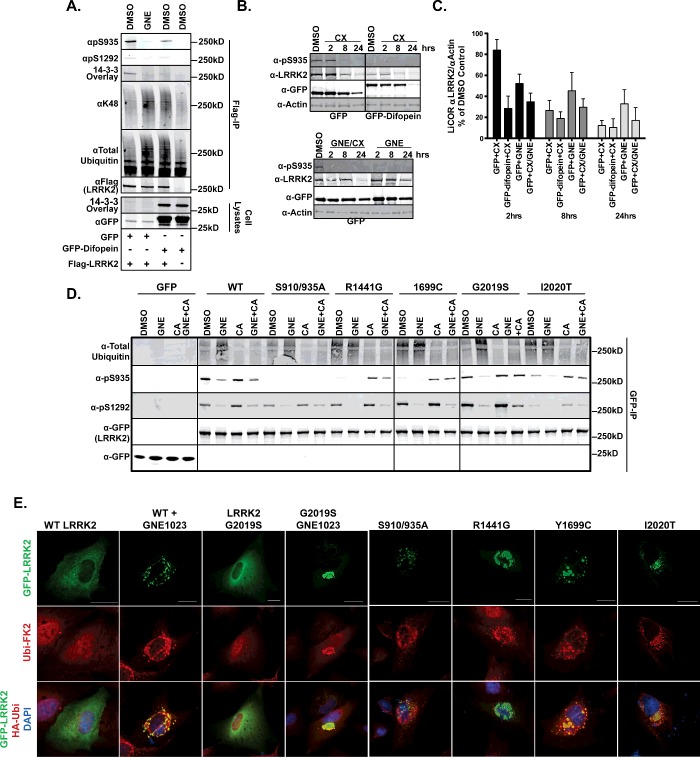
LRRK2 ubiquitination is linked to dephosphorylation of the upstream kinase phosphosites Ser^910/935^ (**A**) Plasmids encoding GFP vector, GFP–difopein and FLAG vector or FLAG–LRRK2 were transfected into HEK293 cells, then treated with DMSO or 2 μM GNE for 24 h. Anti-Flag immunoprecipitations and cell lysates were analysed by immunoblot with anti-FLAG (LRRK2), anti-GFP (GFP or GFP–difopein), anti-ubiquitin (P4D1), Lys48 (D9D5), pSer935 (UDD2), pSer1292 and 14-3-3 overlay for Western (*n*=3). (**B**) Plasmid encoding FLAG–LRRK2 plasmid was co-transfected with GFP-vector or GFP–difopein for 24 h, then cells were treated with 20 μg/ml cycloheximide and/or 2 μM GNE1023 for 2, 8 and 24 h. SDS-soluble (0.5%) lysates were immunoblotted for LRRK2 (N241), pSer935 (UDD2), GFP and actin (*n*=3). (**C**) LI-COR quantified values from (**B**) for 2, 8 and 24 h treatment (α-LRRK2/α-actin) set to untreated control, mean ± S.E.M., (**D**) T-REx cells expressing GFP or GFP–LRRK2 (WT, S910/935A, R1441G, Y1699C, G2019S and I2020T) were treated with 2 μM GNE for 90 min and/or 20 nM calyculin A for 30 min. GFP immunoprecipitates were analysed by immunoblot against pS935 (UDD2) and pS1292, GFP (LRRK2) and ubiquitin (VU1). (**E**) A549 cells were transfected with WT and GFP–LRRK2 (G2019S, S910/935A, R1441G, Y1699C and I2020T). Endogenous ubiquitin was stained with FK2 (red) and green is GFP–LRRK2 and DNA was stained with DAPI (blue). Scale bar is 20 μm. (Larger images provided in Supplementary Figure S4).

Taken together, the data presented above indicate that LRRK2 inhibition results in increased ubiquitination and concomitant decreased protein stability of the protein. A broadly validated phenotype of inhibition of LRRK2 is the dephosphorylation of Ser^910^, Ser^935^, Ser^955^ and Ser^973^ resulting in a loss of LRRK2 binding to 14-3-3 through Ser^910/935^. We postulated that ubiquitination of LRRK2 could be triggered by dephosphorylation of the upstream kinase sites. It is not established how LRRK2 kinase activity signals to a phosphatase or an upstream kinase to regulate Ser^910^, Ser^935^, Ser^955^ and Ser^973^ phosphorylation, if this is indeed the mechanism. To disrupt phosphorylation without direct inhibition of LRRK2, we employed the small peptide 14-3-3 binding inhibitor, difopein [39,66], which we fused to GFP as a gene synthesized, codon optimized ORF. This fusion generated a competent 14-3-3 binding protein shown by 14-3-3 overlay of cell lysates (Figure 4A, bottom panel). Co-expression of FLAG–LRRK2 in the presence of GFP–difopein, but not GFP, caused LRRK2 to become dephosphorylated at Ser^935^, but not Ser^1292^. The dephosphorylation of LRRK2 by difopein expression is accompanied by increased LRRK2 ubiquitination to levels similar to GNE123 treatment (Figure 4A).

Difopein expression induces dephosphorylation of the upstream kinase sites of LRRK2, just as kinase inhibition causes dephosphorylation (Figure 4A) [66]. This allows us to determine the ramifications of dephosphorylation without perturbing kinase activity. We asked if dephosphorylation of LRRK2 by difopein expression would result in decreased LRRK2 stability similar to inhibitor treatment. We compared the stability of LRRK2 co-expressed with GFP or GFP–difopein in the presence of cycloheximide over time. The stability of FLAG–LRRK2 co-expressed with GFP was decreased with cycloheximide, GNE1023 and co-treatment, as with that of GFP–LRRK2 in Figure 1(B) (Figure 4B). We found that difopein expression induced LRRK2 dephosphorylation and decreased the protein steady state levels by 50% compared with GFP alone (Figure 4B) which rapidly decrease in the presence of cycloheximide. These data indicate that the dephosphorylation of LRRK2 at Ser^935^ is sufficient for the ubiquitination and degradation of LRRK2.

Our data from two different approaches of kinase inhibition and difopein expression suggest dephosphorylation and ubiquitination of LRRK2 are linked. In addition to LRRK2 inhibition, LRRK2 is found dephosphorylated at Ser^910/935/955/973^ in N1437H, R1441C/G and Y1699C and I2020T PD-related mutations. This presents an alternate way to investigate the role of dephosphorylation in the ubiquitination of LRRK2. We recently reported that PP1 could mediate the dephosphorylation of LRRK2 and that this could be blocked by treatment with calyculin A, restoring LRRK2 phosphorylation at Ser^910/935/955/973^ after kinase inhibition and in PD mutants [50]. We therefore employed phosphatase inhibition along with LRRK2 kinase inhibition to study the dynamic dephosphorylation of LRRK2 in various mutant alleles. We examined LRRK2 immunoprecipitates from HEK293 T-REx cells expressing GFP, GFP–LRRK2 WT or LRRK2–[S910/935A, R1441G, Y1699C, G2019S and I2020T] mutants treated with GNE1023, calyculin A or both inhibitors (Figure 4D). We found that WT or LRRK2–[G2019S] are similarly phosphorylated at Ser^935^ and exhibit increased ubiquitination after inhibition to similar levels. The increase in LRRK2–[G2019S] kinase activity is revealed by pSer1292 which is also diminished by inhibitor treatment. Co-treatment of calyculin A blocked the inhibitor induced dephosphorylation of the upstream kinase sites [47] but not Ser^1292^ which is accompanied by the reversion of LRRK2 to a lower ubiquitinated species in WT and G2019S LRRK2 (Figure 4D).

LRRK2 R1441G and Y1699C have increased kinase activity shown here by increased Ser^1292^ phosphorylation [14,34,47], but along with I2020T are also dephosphorylated at Ser^935^ [18,47], shown here in Figure 4(D) as well. We found that these mutants exhibit more basal ubiquitination than WT and LRRK2 G2019S. Calyculin A is also able to revert R1441G, Y1699C and I2020T mutants to the phosphorylated state [47,50] which we show is now a minimally ubiquitinated species (Figure 4D). Treatment of cells expressing R1441G, Y1699C and I2020T mutants with GNE1023 did not increase the ubiquitination levels above the already enhanced levels in untreated cells. However, calyculin A treatment was able to restore LRRK2 phosphorylation in R1441G, Y1699C and I2020T mutants with concomitant lower ubiquitination levels of LRRK2. Additionally, phosphatase inhibitor treatment was also able to overcome PD mutation and GNE1023 inhibition to cause decreased ubiquitination of LRRK2 and increased pSer935 (Figure 4D).

We next sought to establish whether LRRK2, inhibited LRRK2 or pathogenic PD-related mutations R1441G, Y1699C and I2020T co-localize with ubiquitin in cells correlative with our biochemical analyses in Figure 4(D). LRRK2 co-localization with ubiquitin has been previously observed for some pathogenic mutations [34,64]. We compared co-localization of endogenous ubiquitin and transiently expressed GFP–LRRK2 and LRRK2 [G2019S] in the presence and absence of GNE1023, as well as PD-related variants of LRRK2 [S910/935A, R1441G, Y1699C and I2020T]. We found little co-localization of ubiquitin with LRRK2 or G2019S LRRK2 probably reflective of the basal state of low ubiquitination of these proteins, which are phosphorylated at the upstream kinase sites. The S910/935A mutant showed co-localization with ubiquitin at discrete cytoplasmic locales known to be caused by mutation or dephosphorylation of Ser^910/935^ [16,17,50], even though this protein shows levels of ubiquitination similar to WT and G2019S LRRK2 (Figure 4D). However, after LRRK2 inhibition and in PD-related R1441G, Y1699C and I2020T mutations, we found co-localization of endogenous ubiquitin with LRRK2 in discrete cytoplasmic locales (Figure 4E).

The S910/935A mutant does not exhibit increased basal ubiquitination, but showed increased ubiquitination after GNE1023 treatment, probably due to the constitutive presentation of the unnatural dephosphorylated LRRK2. This shows that dephosphorylation of LRRK2 is a driver of ubiquitination, but this is not exclusive and LRRK2 kinase activity also might play a role in ubiquitination. However, this does not uncouple dephosphorylation of LRRK2 from the ubiquitination of the S910/935A mutant because calyculin A prevents the inhibitor-induced ubiquitination of this mutant. In a series of transient expression experiments, we examined the ubiquitination of LRRK2 triple mutations of S910/935A with R1441G, G2019S and S1292A and double mutations of S1292A and G2019S in response to GNE1023 (Supplementary Figure S2). We found that S910/935A did not alter the basal or kinase inhibitor induced ubiquitination of S1292A or G2019S compared with single mutants alone. Further, these alanine substitutions did not reduce the enhanced basal ubiquitination of the R1441G mutant. S1292A, G2019S and the combination mutant S1292A/G2019S did not change the ubiquitination from WT, Supplementary Figure S2. These results coupled with our difopein observations above indicate that LRRK2 dephosphorylation is a signal for LRRK2 ubiquitination. Cumulatively, these data show that LRRK2 is ubiquitinated in response to dephosphorylation of the upstream kinase phosphorylation sites. The diverse biology of LRRK2 upstream kinase site phosphorylation has uncovered a mechanism for LRRK2 degradation in pathogenic conditions. This dephosphorylation/ubiquitination cycle is yet another complexity added to the regulation of LRRK2.

## DISCUSSION

Dephosphorylation of the LRRK2 upstream kinase phosphorylation sites is a reliable readout of LRRK2 inhibition [16,17,33]; however, there is little understanding of how this mechanistically affects LRRK2 function. Loss of phospho-serine-dependent 14-3-3 binding has been implicated in altered LRRK2 interaction with ARHGEF7 [67] and a lack of release in exosomes [66]. A currently proposed model is that LRRK2 kinase activity could potentiate a *trans-*acting kinase activity or repress a phosphatase activity toward the upstream kinase phosphosites. Indeed, the inhibitor of nuclear factor kappa-B kinases (IKKs) [68] and casein kinase 1 (CK1) [67] have been identified as upstream kinases and PP1 has been implicated as a physiological phosphatase for the upstream kinase sites [50]. In the present study, we provide novel biochemical insight into the functional significance of LRRK2 phosphorylation by demonstrating a clear inverse relationship between LRRK2 upstream kinase phosphorylation sites and LRRK2 ubiquitination status in expression systems and on endogenous LRRK2. This places LRRK2 in the growing class of molecules and pathways regulated by the interplay of phosphorylation and ubiquitination [69].

The above study set out to understand the relationship between decreased kinase activity and decreased protein levels of LRRK2. We provide several lines of evidence that dephosphorylation of LRRK2, an established result of kinase inhibition, induces the ubiquitination and partial degradation of the protein. First, we show that inhibition decreases the half-life of LRRK2 in a kinase activity-dependent manner in kidney, lung and neuronal cell lines and in lung, kidney and brains of aged mice. Second, we found that inhibition of LRRK2 causes ubiquitination at least via Met^1^/Lys^48^/Lys^63^ linkages. Finally, we demonstrate that the dephosphorylation of LRRK2, through kinase-dependent and -independent mechanisms, is a trigger for ubiquitination.

A potential new therapeutic avenue for LRRK2 genetic PD is to develop a kinase inhibitor that reduces mutant-induced increases in kinase activity [70,71]. LRRK2 is highly expressed in lung, kidney and spleen in the periphery and since most patients have only one mutant allele, it is critical that we identify the molecular consequences of inhibiting the protein. Inhibition of LRRK2 decreases the stability of the protein in a kinase activity manner. When exposed to inhibitor, WT LRRK2, but not an inhibitor-resistant mutant [A2016T], is less stable in cells. Endogenous LRRK2 is also less stable in tissue culture models and in aged mice. LRRK2 stability is decreased in mouse kidney and lung and in NHP kidney when dosed with two similarly potent LRRK2 inhibitors GNE0877 and GNE7915 [33]. Our data are in line with these findings, except that we found diminished LRRK2 protein levels in brain, lung and kidney. Inhibitor effects between tissues and species might be attributed to species specific sensitives to inhibitor and/or different tissue pharmacokinetics of the compound.

LRRK2 can be captured with a steady state of ubiquitin modification; however we show that inhibition of LRRK2 kinase activity increases the total ubiquitination of the molecule in a variety of cell and tissue types (Figure 2). In SH-SY-5Y cells expressing a GFP–LRRK2, we found that inhibitor-induced degradation is dependent on the proteasome but not autophagy, implicating the ubiquitin–proteasome pathway in degradation of a pool of LRRK2. LRRK2 has the potential to be ubiquitinated through multiple linkage types which probably serve as degradation signals (Lys^48^) and/or for signalling (Met^1^, Lys^63^), Figure 3 [63,72,73]. Further, driving linkage-specific ubiquitination in cells also promotes LRRK2 accumulation in cytoplasmic inclusions, similar to what is seen with dephosphorylation of the upstream kinase sites and with other neurological disease proteins [65].

Ser^910/935^ are the probable upstream kinase phosphorylation sites that serve as the signalling switch; blocking 14-3-3 binding to these sites by difopein expression allows LRRK2 to become dephosphorylated by ‘uncapping’ the sites and this induces increased ubiquitination. LRRK2 inhibitor induced dephosphorylation and subsequent ubiquitination is blocked by co-treatment with calyculin A, a PP1 and PP2 inhibitor (Figure 4). The LRRK2 dephosphorylation/ubiquitination cycle is tilted toward the ubiquitinated state in PD mutants R1441C, Y1699C and I2020T since they are dephosphorylated at the upstream kinase sites, which is reversed by calyculin A (Figure 4). Endogenous ubiquitin co-localizes with LRRK2 cytoplasmic accumulations associated with LRRK2 dephosphorylation caused by kinase inhibition and PD-related mutations R1441C, Y1699C and I2020T (Figure 4E). Taken together, these data suggest a direct role for dephosphorylation of LRRK2 in its ubiquitination. Though we observed some ubiquitin co-localization with LRRK2 S910/935A phosphomutant (Figure 4E), we found it is not more highly ubiquitinated in the basal state (Figure 4D), perhaps probably due to expression of this unnatural mutant. S910/935A is still deubiquitinated by co-treatment of calyculin A and LRRK2 inhibitor, similar to WT and G2019S LRRK2, further implicating phosphatase activity in the ubiquitination of LRRK2 (Figure 4D). Since we are able to reverse LRRK2 ubiquitination with calyculin A, we suggest that dephosphorylation of LRRK2 by dynamic means, such as after kinase inhibition, PD mutation or difopein expression is sufficient for increased LRRK2 ubiquitination and degradation.

**Figure 5:**
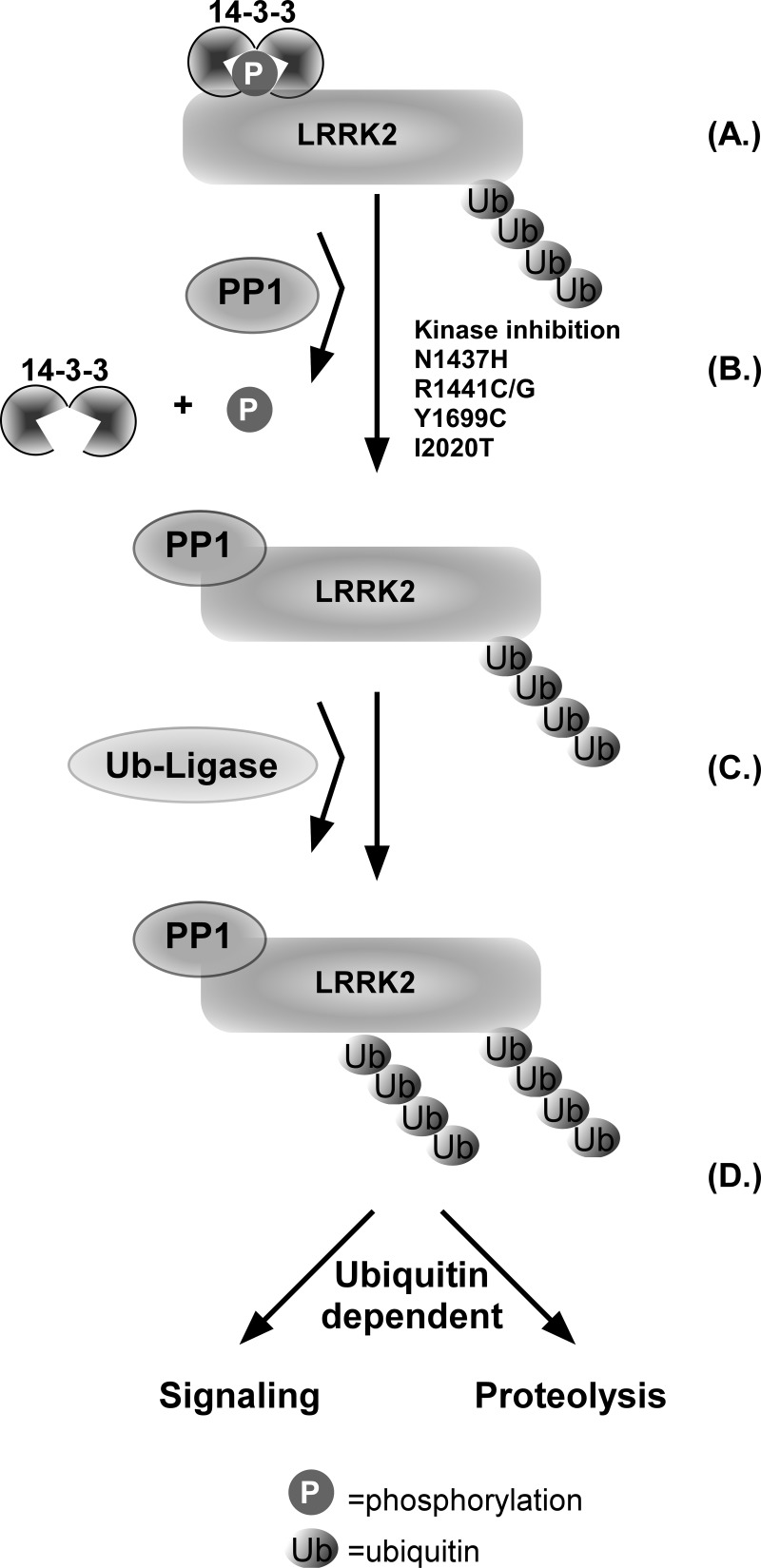
Dephosphorylation of LRRK2 promotes its ubiquitination (**A**) LRRK2 probably exists in a basal ubiquitinated (Ub) and phosphorylated (P) state that is bound to 14-3-3 proteins. (**B**) After kinase inhibition or in pathogenic PD-related mutations N1437H, R1441C, Y1699C and I2020T, a protein such as phosphatase 1 (PP1) is recruited to dephosphorylate LRRK2 causing loss of 14-3-3 binding. (**C**) Dephosphorylation of LRRK2 promotes the addition of ubiquitin to LRRK2 by a ubiquitin ligase. (**D**) This leads to degradation or potentially differential signalling of LRRK2 via ubiquitin linkages.

These data support a model depicted in Figure 5 that proposes LRRK2 probably exists in a basally phosphorylated and ubiquitinated state (A). After kinase inhibition or in PD-related mutations, phosphatases (perhaps through inactivating LRRK2 activity) are recruited through an unknown mechanism to dephosphorylate LRRK2 (B). Our data support that dephosphorylation of the upstream kinase sites leads to ubiquitination of LRRK2 (C). This leads to degradation or signalling of LRRK2 via alternate linkages (D). It stands to reason that kinases phosphorylate LRRK2 which leads to deubiquitination by DUBs to restore the basal state, however this remains to be tested. This has broad implications for the downstream function and signalling of LRRK2, which we show includes decreased stability of total LRRK2 protein and yet to be characterized ubiquitin-dependent signalling complexes.

During LRRK2 inhibitor-based treatment regimens, full ablation of kinase activity with LRRK2 inhibitors would lead to ubiquitinated LRRK2 and decreased LRRK2 protein levels causing similar defects found in peripheral tissues of LRRK2 kinase inactive mutant and LRRK2 KO animals. We have identified a phosphorylation, ubiquitination and degradation cycle as a crucial downstream effect of the well-characterized inhibitor-induced acute dephosphorylation of LRRK2. This presents mechanistic insight for on-target liability [33] and a likely obstacle to LRRK2 inhibitor-based therapeutics. These results indicate that molecules selective for mutant forms of LRRK2 or molecules that are highly selective but low-affinity inhibitors should be evaluated. In the future, it will be necessary to define the ubiquitination linkage types on LRRK2 under various pathogenic conditions. There is probably a diversity of outcomes from LRRK2 ubiquitination. Dephosphorylation of LRRK2 might not only direct the degradation of the protein, but through alternate ubiquitin lysine linkages, the signalling functions of dephosphorylated LRRK2 are likely to be different. This is already evident in some pathogenic mutations where we have previously shown that LRRK2 R1441G, Y1699C and I2020T are dephosphorylated at Ser^910/935/955/973^ and bind more PP1 in cells, showing different protein complexes from WT LRRK2. By exploring the consequences of LRRK2 inhibition, we elucidated dephosphorylation of the upstream kinase sites (Ser^935^) as a mechanistic switch to alter its ubiquitination and downstream stability and function. Additionally, identifying the ubiquitin ligases and DUBs that act on differentially phosphorylated LRRK2 will further elaborate on the mechanisms of its regulation and could serve as novel targets for PD drug discovery.

## Abbreviations

CI: confidence interval
CK: casein kinase
DMEM: Dulbecco's Modified Eagle's medium
DSTT: Division of Signal Transduction Therapy
DUB: deubiquitinase
HA: haemagglutinin
HEK: human embryonic kidney
IKK: inhibitor of nuclear factor kappa-B kinase
KO: knockout
LDS: lithium dodecylsulfate
LRRK2: Leucine Rich Repeat protein Kinase 2
NEM: N-ethylmaleimide
NHP: non-human primates
PD: Parkinson's disease
Roc/COR: Ras of complex/C-terminal of ROC
SOD: superoxide dismutatase
WT: wild-type
